# Role of selected cytokines in the etiopathogenesis of intraventricular hemorrhage in preterm newborns

**DOI:** 10.1007/s00381-016-3217-9

**Published:** 2016-08-19

**Authors:** Dawid Szpecht, Katarzyna Wiak, Anna Braszak, Marta Szymankiewicz, Janusz Gadzinowski

**Affiliations:** 1 Chair and Department of Neonatology, Poznan University of Medical Sciences, Poznan, Poland; 2 Department of Neonatology, Karol Marcinkowski University of Medical Sciences in Poznan, ul. Polna 33, Poznań, Poland

**Keywords:** Intraventricular hemorrhage, Proinflammatory cytokines, Premature birth

## Abstract

Proinflammatory cytokines are essential mediators and indicators of an inflammatory process occurring in the body. Their physiological role is to stimulate the immune response, yet their excessive propagation and interaction with cells outside the immune system may be linked to the risk of organ damage. This is specifically important in the case of immature tissues of fetuses and prematurely born infants. Analysis of the concentrations of specific cytokines in different compartments makes it possible to assess the risk of premature birth, preterm rupture of the membranes, and to determine an existing intrauterine infection. The purpose of this paper is to summarize the existing research concerning the relationships between the concentrations of specific proinflammatory cytokines in different compartments (maternal blood serum, amniotic fluid, umbilical cord blood, arterial and venous blood, and cerebrospinal fluid of the newborn) and the risk of intraventricular hemorrhage (IVH) and the degree of its severity. The paper takes also into account the assessment of the usefulness of cytokines as biomarkers for IVH and its complications (posthemorrhagic hydrocephalus, white matter injury).

Dynamic development of perinatal medicine and neonatology has contributed to increased survival rates among prematurely born infants, leading at the same time to a relative increase in the number of multi-organ complications occurring in these babies. Intracranial, mainly intraventricular hemorrhages (IVHs), resulting from the immaturity of brain structures, represent one of the most common pathologies in premature infants. The incidence of this pathology is inversely proportional to gestational age. There are four degrees of IVH severity, graded I–IV [21]. Bleeding graded I and II is often self-limiting. Higher grade hemorrhages result in higher risk of neurodevelopmental delay; they are also the main cause of cerebral palsy and increased perinatal mortality rates in this group of infants [7, 20]. It is estimated that IVH occurs in as much as 20% of infants born before 32nd week of gestation [4].

The etiopathogenesis of IVH is complex. Besides gestational age (below 32 weeks) and body weight at birth (less than 1500 g), there are also other IVH risk factors identified, including absence of prenatal steroid therapy in women at risk of premature delivery, early clamping of the umbilical cord (less than 30 s after birth), symptoms of intrauterine infection in the mother and the newborn, and labor and delivery complicated by bleeding or perinatal hypoxia. The group of infants with an elevated IVH risk includes also newborns with intrauterine growth retardation or those who in the first days of their life were treated with crystalloids (bolus 10–15 ml/kg) and/or catecholamins for hypotension and NaHCO_3_ for acidosis. IVH risk group includes also newborns with blood clotting disorders, thrombocytopenia, hypoglycemia, pneumothorax, lung bleeding, and those transported from another hospital [2, 5]. Moreover, nowadays, an increasing attention is being paid to genetic factors in the development of IVH; however, it has only been confirmed by few studies so far.

The purposes of the paper are to review and summarize the results of studies to date concerning the impact of the concentration of specific proinflammatory cytokines measured in the mother, the fetus, and the newborn on the risk of IVH and to discuss genetic factors whose involvement in IVH pathogenesis has been confirmed in the highest number of reports and for which being a carrier plays an important role in their pathophysiology [31].

## Interleukin-1 (IL-1)

Interleukin-1 (IL-1) plays a significant role in Th1 cell response. It is produced by activated macrophages as well as by cells outside the immune system such as keratinocytes. It stimulates the production of other proinflammatory cytokines, such as interferon gamma (INF-y), interleukin-6 (IL-6), and tumor necrosis factor (TNF). IL-1 forms a cytokine family composed of more than 10 subtypes: IL-1α, IL-1β, IL-1y (IL-18), IL-1e, IL-1d, IL-1Ra, and IL-1H, of which IL-1β is the most responsible for the proinflammatory effect [33].

Increased levels of IL-1 accompany various inflammatory processes, which can increase the risk of IVH. A number of earlier studies indicated the risk of IVH in infants born prematurely from pregnancies complicated by chorioamnionitis [19, 22]. The research by Viscardi et al. [34], where association between the concentration of inflammatory markers in umbilical cord or vein blood of an infant and the risk of neurological abnormalities was investigated, has not confirmed this relationship. The sample comprised 276 infants born before 33rd week of gestation with very low body weight at birth. Significantly elevated levels of IL-1 in the blood serum and cerebrospinal fluid (CSF) of these infants were associated with intrauterine infections, compared to infants born from pregnancies with no inflammatory response complications. However, a straightforward relationship between intrauterine infection/elevated IL-1 levels and the risk of abnormal neurologic outcome, including IVH, was not observed. Similar results were found by Kaukola et al. [15] who investigated the relationships of chorioamnionitis and levels of cord blood’s proinflammatory cytokines with the presence of neurological pathologies registered by cranial ultrasound scan (CUS) by fourth week of life and neurological outcome assessed at the corrected age of 24 months. The sample comprised 78 extremely low birth weight infants born before 32nd week of gestation. They were classified into four groups, following the results of histological tests of placenta (isolated histologic chorioamnionitis (HCA), isolated placenta perfusion defect, HCA and perfusion defect, no HCA or perfusion defect). No significant differences between these groups were observed for IL-1 blood serum levels.

Damage to the newborn’s brain may follow an intrauterine infection that induced maternal and fetal immune response, both systemic and localized to central nervous system (CNS). Microorganisms and the mediators they produce stimulate mononuclear cells to produce IL-1, and its elevated levels increase the permeability of the blood-brain barrier, thus facilitating the passage of these substances into the neonate’s brain tissue [35]. Viscardi et al. [33] investigated associations between the intrauterine presence of *Ureaplasma parvum* and *Ureaplasma urealyticum* and the occurrence of IVH in a sample of 13 very low birth weight neonates born prematurely on the 32nd week of gestation, of whom 74 had *Ureaplasma* antigens in serum or cerebrospinal fluid. The analysis of both cord and venous serum samples taken together demonstrated significantly higher IL-1 levels in *Ureaplasma* PCR-positive infants, compared to PCR-negative ones. However, the correlation was not present when the analysis was done for cord blood samples only, where increased levels of IL-1 were found not only in 70% of seropositive infants but also in almost half of the seronegative group. Correlation between CSF cytokine levels and *Ureoplasma* sp. infection was not observed. The incidence of IVH was evaluated on the basis of a cranial ultrasound scan. For infants infected with *Ureaplasma* sp., the risk of IVH III–IV increased 2.32-fold. When the group included only the infants with increased serum IL-1 levels, the risk was 5.36 times higher.

As demonstrated in the research into proinflammatory cytokine polymorphisms, genetic factors have an impact on the risk of IVH. A study by Ryckman et al. [24] included 271 very low birth weight infants born before 32 weeks of gestation. Ten genes potentially linked to increased IVH risk were isolated and examined, with specific single nucleotide polymorphisms selected for these candidate genes, based on previous research. A significant correlation was demonstrated only in the case of IL-1β-31 gene polymorphism. Children with the CT genotype or the IL-1β-31 C allele were at a higher risk for IVH I-II and those with the CC genotype or the C allele, for IVH III-IV, compared to children with the TT genotype or the T allele. The IL-1β-31 C allele is associated with an increased production of IL-1β in vivo. This may represent an evidence that IL-1β plays a role in the pathophysiology of perinatal brain injury. Increased levels of IL-1β are observed in the amniotic fluid and/or cord blood of infants with IVH or periventricular leukomalacia (PVL) [6].

Concentration of proinflammatory cytokines may also be useful in predicting the complications of IVH. Following IVH or posthemorrhagic hydrocephalus (PHH), many infants develop white matter injuries (WMIs), resulting probably from the adverse involvement of proinflammatory cytokines. Correlations between concentration levels of specific cytokines and the development of white matter damage have been investigated by Schmitz et al. [28], in the paper cited in a review paper by Stephanie Merhar [20]. The studies show that PHH neonates have increased levels of IL-1β in their CSF, compared to the non-PHH control group, regardless of WMI. Savman et al., whose research is also cited in the paper, studied levels of cerebrospinal fluid cytokines in premature infants with PHH and the correlations of these with periventricular white matter injury, neurodevelopmental impairment, and the need of intervention by insertion of a ventriculoperitoneal shunt. Compared to the control group, increased CSF levels of IL-1β, TNFα, IL-6, and IL-8 were found in preterm infants with PHH. However, a relationship between the levels of these cytokines and white matter lesions was not observed. This would indicate that the presence of cytokines is a reaction to the hemorrhage and does not reflect the severity of brain damage.

## Interleukin 6 (IL-6)

IL-6 is a cytokine with a very wide spectrum of action in the areas including the development of the nervous and hematopoietic systems. It is an early marker of inflammation, a mediator of the acute phase response, and an activator of the immune system, reaching its peak levels as early as 3–4 h after pathogen stimulation. IL-6 levels rise also after mechanical and thermal tissue damage. Its action is bidirectional: both proinflammatory but also antiinflammatory (the latter by acting against TNF-alpha and IL-1). It can cross the blood-brain barrier [13].

Caldas et al. [8] studied IL-6 levels in umbilical cord blood of 125 infants with very low birth weight, while the incidence rate of IVH was assessed through cranial ultrasound scans. No statistically significant associations were found between IL-6 levels and IVH either. No correlations of this kind were found by Bhandari et al. [7] in a study of 116 infants born between weeks 23 and 34 of gestation either. Sorokin et al. [30] also investigated the associations between umbilical cord blood IL-6 levels and the risk of IVH in a group of 400 infants (gestational age at birth 34.8 ± 3.8 weeks). Again, no significant correlation between IL-6 levels (>75 pc) and an increased risk of IVH was observed. These studies confirmed the impact of increased umbilical cord blood IL-6 levels on an increased risk of a preterm delivery.

There is, however, an association between an increased risk of IVH and prolonged premature rupture of membranes (PPROM). The study by Hansen-Pupp et al. [12], concerning correlations between cytokine levels and PPROM, IVH, and WMI, included 74 infants born before 32 weeks of gestation. Higher cord blood levels of IL-6 were observed in the group of infants with maternal PPROM. Their IL-6 level peaked at delivery, while in infants without PPROM, IL-6 peaked in the arterial blood 6 h after birth. It then decreased in both groups and stabilized at 72 h.

In the previously mentioned study by Kaukola et al. [15], umbilical cord blood IL-6 levels were significantly elevated in the group of infants with isolated HCA, compared to the group with placenta perfusion defect and the group with neither HCA nor perfusion defect. HCA, in turn, increased the risk of IVH, mainly grades II and III. No direct association was observed between cord blood IL-6 levels and the development of IVH. Additionally, high level of IL-6 in umbilical cord blood was highly predictive of the risk of premature birth. In another study by Sarkar et al. [25], carried out on a group of 62 infants born before 28 weeks of gestation, HCA was not found to be associated with an increased risk of early onset IVH.

Goldenberg et al. [10] studied the frequency of umbilical cord blood infections with *U. urealyticum* and *Mycoplasma hominis* in preterm 23- to 32-week infants. He also evaluated the associations of these infections with obstetric complications, markers of placental inflammation, and other pathological conditions in the newborn. The study included 351 mother-infant pairs. Bacteria were present in 23 % of infant cord blood cultures which correlated with an increased incidence of intrauterine infection and inflammation among these infants. This was confirmed by an increased level of IL-6, placental bacteria cultures, and placental histology. *Ureaplasma* or *Mycoplasma*-positive infants were at higher risk of developing systemic inflammatory response syndrome (SIRS) and bronchopulmonary dysplasia (BPD), whereas an increased incidence of other neonatal pathologies, such as IVH, respiratory distress syndrome (RDS), or death, was not observed.

The study by Heep et al. [13] investigated postnatal levels of IL-6 in serum and brain injury shown in cranial ultrasound scan and clinical symptoms in infants born before 28th week of gestational age. The sample comprised of 88 infants grouped according to the peak IL-6 level within 12 h of postnatal age: <97 pc (≤100 pg/ml) and >97 pc (>100 pg/ml) of healthy infants. IVH was present significantly more often (63 % of infants) in the group with IL-6 levels above 100 pg/ml. In the group where IL-6 was below the concentration threshold, IVH occurred in 38 % of infants. Higher IL-6 levels were associated not only with a higher risk of IVH but also with increased severity and higher neonatal morbidity at <28 days after birth. These results allow the use of IL-6 as an early marker of bleeding.

Pathogenesis of IVH may involve interactions between proinflammatory cytokines and the coagulation system. Vitamin K-dependent coagulation factors and infant serum IL-6 levels were studied by Poralla et al. [23]. The study sample comprised 132 extremely premature infants who were divided into two groups: with IL-6 above and below 100 pg/ml. Increased IL-6 levels were associated with decreased factor VII activity and increased concentration of fibrinogen. These factors may play a role in IVH pathogenesis by causing systemic and local hemodynamic changes and by damaging the endothelium of cerebral vessels.

Research by Viscardi et al. [34] demonstrated that IL-6 levels in blood serum and cerebrospinal fluid of infants affected by maternal intrauterine infection were more often significantly elevated, compared to those of non-affected babies. A correlation was demonstrated between infant venous blood serum IL-6 levels and the presence of abnormalities in CUS, but only in infants born after 28th week of gestation. With regard to CSF, a similar correlation was observed only in infants born before or in week 28 of gestational age.

The purpose of a research conducted by Satar et al. [26] was to search for the association of the levels of proinflammatory cytokines in maternal and cord blood at birth with neonatal morbidity and mortality in the group of infants with PPROM. The study group included 42 mother-infant pairs with PPROM, and the control group comprised 41 pairs without PPROM. All infants were born between weeks 29 and 35 of gestation. Maternal serum levels of IL-6 in the group with PPROM were significantly higher than in the control group, while a similar correlation for cord blood was not found. Infants born after PPROM had a higher risk of IVH, but a direct association between increased IL-6 concentration and the development of IVH was not demonstrated.

Sorokin et al. [29] investigated the association of maternal serum IL-6 levels with the risk of premature birth and IVH development in infants. Samples were taken between weeks 24 and 32 of pregnancy from 475 patients who carried a risk of premature delivery and had intact membrane and who had received glucocorticoids (betamethasone) 7–10 days before sample taking. Mothers of infants born before week 32 had increased levels of IL-6 in blood serum (>90 pc).

Göpel et al. [11] tested an earlier hypothesis that the IL-6-174GG genotype is associated with increased susceptibility to sepsis and the 174CC genotype is more common in preterm infants with severe IVH. The study included 1206 prematurely born infants with birth weight below 1500 g. No significant correlations were demonstrated between different IL-6-174 genotypes and grade IV IVH, leukomalacia, or infant death.

## Interleukin 8 (IL-8)

IL-8 is a proinflammatory cytokine produced mainly not only by macrophage cells but also by smooth muscle cells and endothelial cells. It is also known as “neutrophil chemotactic factor,” which describes one of the primary functions of this cytokine. Besides, it also induces the process of phagocytosis by the cells that arrived at the site of an inflammation. Carlo et al. demonstrated the role of IL-8 in brain damage during the processes of ischemia reperfusion, anoxia, and traumas. Activated neutrophils accumulate in cerebral blood vessels, thus obstructing microvascular perfusion and causing a release of various other proinflammatory mediators [9].

The associations of IL-8 levels in the newborn’s blood with the occurrence of IVH and IVH-associated white matter lesions (WMIs) were studied by Leviton et al. [18]. One hundred twenty-three infants born before week 28 of gestation who had IVH but no WMI were compared to a group of neonates with both IVH and WMI and a control group of 677 healthy infants. Of all investigated cytokines, elevated levels of IL-8 persisting for more than 1 day were most significantly associated with isolated IVH as well as both IVH and WMI, compared to the control group.

Increased levels of IL-8 in cord blood are associated with PPROM and HCA, histologically diagnosed postpartum. Both these conditions increase the risk of infant IVH, but no direct association between IL-8 levels and a development of IVH was demonstrated [15, 26].

Krediet et al. [16] investigated the relationship between respiratory distress syndrome (RDS) and the risk of early vs. late IVH (in the first 12 h of life vs. after 12 h). The levels of proinflammatory cytokines in arterial blood were measured in 114 infants born before week 32 of gestation of whom 67 were diagnosed with RDS. Increased level of IL-8, induced by RDS-related inflammation, increased the risk of early IVH only. After 12 h of life, the level of IL-8 was still correlated with RDS but had no impact on the development of late IVH.

Maternal intrauterine inflammation results also in an inflammatory response in the infant’s central nervous system. In their research, Laborada and Nesin [17] evaluated whether exposure to chorioamnionitis and fetal inflammatory syndrome was reflected in increased concentrations of selected proinflammatory cytokines (TNF-alpha, IL-6, and IL-8) in the cerebrospinal fluid of premature and term infants. The results demonstrated a significant increase in IL-8 concentrations in the CSF of neonates exposed to chorioamnionitis and maternal/fetal inflammation.

## Interleukin-18 (IL-18)

IL-18 is a proinflammatory cytokine that may mediate injuries to the central nervous system. IL-18 is expressed in astrocytes and microglia, and its receptor is found on astrocytes in the periventricular area of the brain. Increased cord blood levels of IL-18 are correlated with white matter damage and subsequent development of cerebral palsy. Increased levels of IL-18 and IL-1β in cerebrospinal fluid are associated with posthemorrhagic ventricular dilation [3].

## Tumor necrosis factor (TNF-alpha)

TNF-alpha is a protein involved in the activation of the immune system. This cytokine is produced mainly not only by activated macrophages but also by a variety of other cell types such as lymphoid cells, mast cells, endothelial cells, cardiomyocytes, adipocytes, connective tissue cells, and neurons. It impacts various organ systems, with its biological functions overlapping the functions of IL-6. It is a chemoattractant for neutrophils and stimulates macrophages for phagocytosis and production of both IL-1 and prostaglandin E2 (PGE2—which plays a role in the etiopathogenesis of preterm birth). It can cause damage to microcirculatory endothelium of the central nervous system, increasing the risk of vessel rupture and IVH occurrence [31].

TNF may participate in the pathophysiology of periventricular injury in several ways: by inducing fetal hypotension and brain ischemia, by stimulating the production of a tissue factor which then can activate the coagulation system and lead to necrosis of white matter, by stimulating the production of platelet-activating factor which can damage cell membranes and cause direct brain cell damage, and by a direct cytotoxic effect on oligodendrocytes.

Microbes and proinflammatory cytokines in peripheral blood may, after crossing the blood-brain barrier which has been weakened by inflammation, stimulate microglia to produce proinflammatory cytokines. They, in turn, can activate the proliferation of astrocytes and production of TNF-alpha which can cause damage to oligodendrocytes, the cells responsible for the deposition of myelin. Yoon et al. [35] tested post mortem 17 brains of infants diagnosed with PVL and 17 controls. The expression of TNF-alpha, IL-1beta, or IL-6 was found in 88 % of cases with PVL and in only 18 % of non-PVL cases. Cytokines were present mainly in hypertrophic astrocytes and microglial cells located in and around the necrotic areas.

Vinukonda et al. [32] asked whether cyclooxygenase-2, its derivative prostaglandin E2, prostanoid receptors, and proinflammatory cytokines (IL-1, TNF-alpha) were elevated in intraventricular hemorrhage and if their suppression confers neuroprotection. They showed that cyclooxygenase-2, prostanoid receptor-1, or tumor necrosis factor-α inhibition reduced inflammatory cell infiltration, apoptosis, neuronal degeneration, and gliosis around the ventricles of studied pups with intraventricular hemorrhage. Moreover, prostanoid receptor-1 and tumor necrosis factor-α were downstream to cyclooxygenase-2 in the inflammatory cascade induced by intraventricular hemorrhage. On the other hand, cyclooxygenase-2-inhibition or suppression of downstream molecules prostanoid receptor-1 or TNF-alpha might constitute neuroprotective strategy for minimizing brain damage in premature infants with intraventricular hemorrhage. Conclusions from studies carried out on animal models may contribute to similar methods being applied in prematurely born infants with IVH, resulting in reduced mortality and neurological complications in this group.

Viscardi et al. [34] investigated the association between the concentration of inflammatory markers in CSF and the risk of neurological abnormalities detected in CUS. The sample comprised 276 infants born before week 33 of gestation with very low birth weight (<1501 g). Levels of TNF-alpha found in cerebrospinal fluid of infants with intrauterine infections did not differ significantly from those found in infants born from pregnancies not complicated by infection. At the same time, a positive correlation of TNF-alpha CSF levels and abnormalities shown in CUS was demonstrated, but only in infants born before or in week 28 of gestation. It is not possible, however, to conclude unambigously whether increased TNF-alpha levels were a cause or an effect of damage to the central nervous system. The ultrasound scan was done after measuring cytokine levels in CSF.

In the case of TNF-alpha, genetic factors may play a role in the risk of IVH. An important polymorphism occurs at position 308 in the TNF-alpha promoter region. Three variants exist: homozygotic GG or AA and heterozygotic AG. The A allele is responsible for strong and allele G for weak gene expression in vitro. Previous research demonstrated an increased risk of IVH in neonates with the TNF alfa-308 A allele. In a study by Adcock et al., done on a sample of 178 newborn infants, 30% carried the A allele: 25% were heterozygotic (AG) and 5% were homozygotic (AA). Homozygotic GG variant was found in 70% of infants. Carriers of the A allele showed an increased risk for IHV (grades I to IV), but when the group considered included only grades III and IV, the association became not significant [1]. Heep et al. [14] did a retrospective study on a sample of 27 infants born before week 32 of pregnancy who had severe IVH and a control group of 102 healthy infants, with no IVH and born after week 32. Differences in the TNF-alpha 308 expression were not observed between the groups.

To summarize, the role of cytokines in pathogenesis of IVH is complex, and the role of proinflammatory cytokines is not fully known. It seems that concentrations of specific cytokines in different compartments make it possible to assess the risk of premature birth, preterm rupture of the membranes, and to determine an existing intrauterine infection: Increased levels of IL-1 in the neonate’s blood and the presence of certain polymorphisms correlate with an increased risk of IVH in premature infants; in the cerebrospinal fluid, IL-1 may also be a marker of existing consequences of IVH, such as PHHC; increased levels of IL-6 may be used for the assessment of the risk of premature delivery and intrauterine infection; measurement of the levels of TNF-alpha and in the future administration of TNF-alpha inhibitors may contribute to reducing adverse neurological outcomes in infants who developed IVH. We suggest that further analysis of the role of polymorphism of selected cytokines should be performed. This could probably make it possible to determine the group of newborns who are specifically at risk of developing IVH in the perinatal period (Table 1).

**Table 1:** The overall significance of the selected cytokines in the pathogenesis of abnormalities in central nervous system

Cytokine	Compartment	Clinical significance and association with central nervous system abnormalities
IL-1β	Infant’s vein blood serum	No association with increased risk of abnormal neurological outcome (including IVH) [34]
	Cerebrospinal fluid	Association with increased risk of PHH, irrespective of coexistence of WMI [28]. Association with increased risk of PHH, but not with WMI [27]. No association with increased risk of abnormal neurological outcome (including IVH) [34]
IL-6	Maternal vein blood serum	No association with increased risk of IVH [26]
	Umbilical cord blood	No association with increased risk of IVH [7, 8, 10, 15, 30]
	Infant’s vein blood serum	Association with increased risk of IVH, its severity, and higher neonatal morbidity [13]. Association with abnormalities in CUS only in infants born after or in week 28 of gestational age [34]
	Cerebrospinal fluid	Association with increased risk of PHH, but not with WMI [27]. Association with abnormalities in CUS only in infants born before or in week 28 of gestational age [34]
IL-8	Umbilical cord blood	No association with IVH [15, 26]
	Infant’s vein blood serum	Association with isolated IVH and both IVH and WMI [18]
	Cerebrospinal fluid	Association with increased risk of PHH, but not with WMI [27]
IL-18	Cerebrospinal fluid	Association with posthemorrhagic ventricular dilatation [3]
TNF-α	Cerebrospinal fluid	Association with abnormalities in CUS only in infants born before or in week 28 of gestational age [34]. Association with increased risk of PHH, but not with WMI [27]
